# Three-Dimensional Thoracic Aortic Remodeling Associated with Kommerell Diverticulum Enlargement in Adults with a Right-Sided Aortic Arch: A Quantitative CTA Study

**DOI:** 10.3390/jcdd13090427

**Published:** 2026-09-01

**Authors:** Xiaolong Zhang, Jiehua Li, Hao He, Xin Li, Chenzi Yang, Chang Shu

**Affiliations:** 1 Department of Vascular Surgery, Wuhan No. 1 Hospital, 215 Zhongshan Avenue, Qiaokou District, Wuhan 430022, China; zhangxiaolong1003@outlook.com; 2 Department of Vascular Surgery, The Second Xiangya Hospital, Central South University, 139 Renmin Road, Changsha 410011, China; 3 Department of Vascular Surgery, Fuwai Hospital, Chinese Academy of Medical Sciences and Peking Union Medical College, Beijing 100037, China

**Keywords:** right-sided aortic arch, Kommerell diverticulum, computed tomography angiography, three-dimensional imaging, aortic remodeling, thoracic endovascular aortic repair

## Abstract

Background: Right-sided aortic arch with Kommerell diverticulum (RAA-KD) has complex three-dimensional anatomy. Methods: We retrospectively analyzed computed tomography angiography data from 65 adults with RAA-KD using centerline-based reconstruction. Patients were stratified by conventional maximal KD diameter (D_KDmax_) into <55 mm (*n* = 38) and ≥55 mm (*n* = 27) groups. Age associations, adjusted group differences, and continuous associations with centerline-derived KD diameter at maximal cross-sectional area (D_10_) were examined. Results: After sex adjustment and false-discovery-rate correction, all aortic diameters increased with age; the ascending aorta, arch, and descending thoracic aorta also elongated, whereas aortic isthmus (L_4_) and KD (L_5_) lengths were not age-associated. After age and sex adjustment and false-discovery-rate correction, diameters of the ascending aorta, aortic arch, and descending thoracic aorta (D_2_–D_8_), as well as KD-related diameters (D_9_–D_12_), remained larger in the ≥55 mm group; L4 was shorter and L5 longer. In continuous models, D_10_ was positively associated with all non-KD diameters, most strongly with the adjacent proximal descending aorta (D_7_), and independently associated with shorter L_4_ (B = −0.548; 95% CI, −0.828 to −0.268) and longer L_5_ (B = 0.639; 95% CI, 0.412–0.866; both *p* < 0.001). Conclusions: RAA-KD demonstrates generalized age-associated thoracic aortic enlargement and localized KD-associated remodeling. Comprehensive three-dimensional assessment may inform evaluation of the adjacent aorta and proximal sealing zone for thoracic endovascular aortic repair.

## 1. Introduction

A right-sided aortic arch (RAA) is an uncommon congenital vascular anomaly that may coexist with an aberrant left subclavian artery (ALSA) arising from a Kommerell diverticulum (KD) [1,2,3,4,5,6,7,8]. Although many adults are asymptomatic, KD enlargement may cause tracheoesophageal compression and life-threatening complications, including aortic dissection or rupture [1,3,9,10,11,12,13]. Accurate characterization of this complex anatomy is therefore fundamental to both surveillance and therapeutic decision-making.

Computed tomography angiography (CTA) provides high-resolution three-dimensional visualization of the aortic arch, branch vessels, and adjacent structures and is central to planning open, hybrid, and thoracic endovascular aortic repair (TEVAR) [14,15,16]. Conventional measurements described by Ota and Idrees remain clinically useful but primarily characterize diverticular size [17,18]. They provide limited information about the geometric relationship between the KD and the adjoining thoracic aorta. Furthermore, measurements made on axial images may be affected by tortuosity and oblique imaging planes.

Published evidence has largely comprised case series, operative cohorts, and studies of KD diameter or short-term natural history [7,8,10,12,13,19]. Quantitative data describing both radial and longitudinal morphology across the thoracic aorta in adults with RAA-KD remain limited. Such information may be clinically relevant because remodeling of the distal arch and proximal descending aorta could alter the length and geometry of the proximal sealing zone used for TEVAR.

This study therefore used centerline-based three-dimensional CTA reconstruction to characterize thoracic aortic morphology in adults with RAA-KD. We evaluated age-associated differences, compared patients with conventional maximal KD diameters below and above 55 mm, and examined continuous associations between KD size and adjacent aortic geometry. We hypothesized that age would be associated with generalized aortic enlargement and elongation, whereas increasing KD size would be associated with localized enlargement and reciprocal changes in aortic isthmus and diverticular length.

## 2. Materials and Methods

### 2.1. Study Design and Patient Identification

This retrospective, cross-sectional, single-center study was approved by the Institutional Review Board of The Second Xiangya Hospital of Central South University. The requirement for informed consent was waived because of the retrospective design. The study is reported in accordance with the Strengthening the Reporting of Observational Studies in Epidemiology (STROBE) statement.

Consecutive adults with RAA-KD who underwent contrast-enhanced CTA between January 2013 and July 2021 were identified through the institutional radiology information system. Patients were excluded if they had an aberrant subclavian artery without KD, an isolated aortic diverticulum without continuity with the subclavian artery, a double aortic arch, congenital heart disease, previous surgical or endovascular intervention involving the aortic arch or KD, an acute aortic syndrome-including dissection, intramural hematoma, or penetrating aortic ulcer-or a thoracic aortic aneurysm unrelated to KD.

Given the rarity of RAA-KD and the retrospective nature of this study, no a priori sample-size calculation was performed. All eligible adult patients identified during the predefined study period were included.

### 2.2. Clinical Data

Electronic medical records were reviewed for age, sex, height, symptoms attributable to tracheal or esophageal compression, hypertension, diabetes mellitus, ALSA stenosis, aortic arch calcification, vertebral artery variants, thyroid hemiagenesis, and an aberrant left brachiocephalic vein. When outpatient clinical information was unavailable in the electronic record, supplementary information was obtained by telephone.

Associated vascular and anatomical anomalies were identified on CTA according to established anatomical descriptions [20,21,22], and aortic arch calcification was recorded as present or absent [23].

### 2.3. CTA Acquisition and Three-Dimensional Reconstruction

CTA scans were carried out at the Second Xiangya Hospital using a third-generation dual-source CT scanner (SOMATOM Definition Force, Siemens Healthcare, Forchheim, Germany), with a high iodinated contrast bolus of 100 mL (370 mg I/mL). The bolus was injected at a rate of 4–5 mL/s and was chased by saline. CTA acquisitions were not ECG-gated and therefore were not synchronized to a predefined cardiac phase. CTA images with a maximum slice thickness of 1 mm were accepted for further processing.

CTA datasets were imported into Materialise Mimics (version 22.0; Materialise NV, Leuven, Belgium). Three-dimensional models of the thoracic aorta were reconstructed using semiautomated thresholding and region-growing segmentation. An aortic centerline was generated automatically and manually adjusted when necessary to ensure anatomic accuracy. Cross-sectional measurements were obtained on planes perpendicular to the centerline, reducing errors related to vascular tortuosity and oblique imaging planes [24].

### 2.4. Measurement Definitions

**Figure 1:**
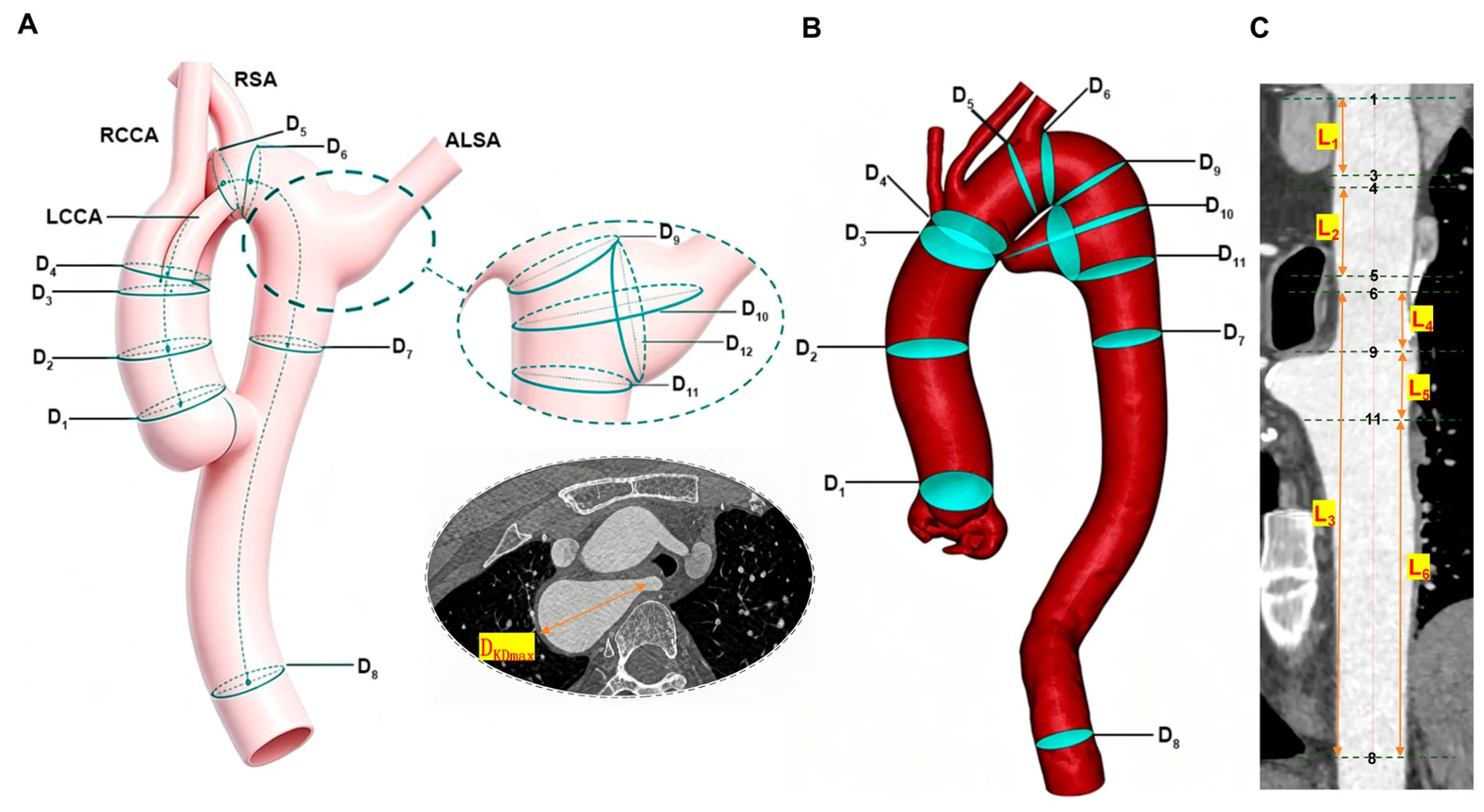
Three-dimensional centerline-based measurements of thoracic aortic morphology in patients with right-sided aortic arch and Kommerell diverticulum (RAA-KD): (**A**) Schematic illustration of the measurement protocol for thoracic aortic diameters. Diameters were measured on planes perpendicular to the aortic centerline at predefined anatomical landmarks. D_1_–D_8_ represent the diameters of the non-KD thoracic aorta, measured at the sino-tubular junction (D_1_), mid ascending aorta (D_2_), proximal edge of the left common carotid artery (LCCA, D_3_), distal edge of the LCCA (D_4_), proximal edge of the right subclavian artery (RSA, D_5_), distal edge of the RSA (D_6_), proximal descending thoracic aorta located 2 cm distal to the KD (D_7_), and descending thoracic aorta at the diaphragmatic hiatus (D_8_). D_9_–D_12_ represent the KD-related diameters measured at the proximal edge of the diverticulum (D_9_), the maximal centerline-perpendicular diameter at the level of the maximal KD cross-sectional area (D_10_), the distal edge of the diverticulum (D_11_), and the orifice of KD (D_12_). The axial CTA image illustrates the conventional maximum Kommerell diverticulum diameter (D_KDmax_), measured from the contralateral aortic wall to the apex of the diverticulum. (**B**) Corresponding D_1_–D_12_ measurement planes on the three-dimensional CTA reconstruction. (**C**) Measurement of longitudinal aortic lengths on curved multiplanar reconstruction along the aortic centerline. L_1_ indicates the ascending aortic length from the sino-tubular junction to the proximal edge of the LCCA; L_2_, the aortic arch length from the distal edge of the LCCA to the proximal edge of the RSA; L_3_, the descending thoracic aortic length from the distal edge of the RSA to the diaphragmatic hiatus; L_4_, the aortic isthmus length from the distal edge of the RSA to the proximal margin of the KD; L_5_, the longitudinal length of the KD from its proximal to distal margin; and L_6_, the descending thoracic aortic length distal to the KD extending to the diaphragmatic hiatus. Abbreviations: KD, Kommerell diverticulum; D_KDmax_, maximal Kommerell diverticulum diameter; LCCA, left common carotid artery; RCCA, right common carotid artery; RSA, right subclavian artery; ALSA, aberrant left subclavian artery. The conventional maximal KD diameter (D_KDmax_) was measured from the contralateral aortic wall to the apex of the diverticulum using multiplanar reconstruction, as described by Idrees et al. [17]. D_10_ was the centerline-derived equivalent diameter calculated from the maximal KD cross-sectional area (A_10_). These measurements represent different geometric constructs and were analyzed as complementary rather than interchangeable measures.

Following established approaches to thoracic aortic measurement [14], cross-sectional areas of non-KD-related aortic segments (A_1_–A_8_) were measured on planes perpendicular to the centerline at the following landmarks: A_1_, the sinotubular junction; A_2_, the mid-ascending aorta; A_3_, the proximal edge of the left common carotid artery (LCCA), representing the distal ascending aorta; A_4_, the distal edge of the LCCA, representing the proximal arch; A_5_, the proximal edge of the right subclavian artery (RSA), representing the distal arch; A_6_, the distal edge of the RSA, representing the aortic isthmus; A_7_, the proximal descending aorta 2 cm distal to the distal edge of KD; and A_8_, the aorta at the level of the aortic hiatus, respectively. Areas of KD-related segments (A_9_–A_12_) were measured at A_9_, the proximal edge of KD; A_10_, the plane of maximal KD cross-sectional area; A_11_, the distal edge of KD; and A_12_, the orifice of KD, respectively (Figure 1A) [25].

Each cross-sectional area (*A*) was converted to an equivalent circular diameter (D_1_–D_12_) using the following equation:

$$D=2*\sqrt{\;A/\pi }$$

where *D* is the equivalent circular diameter and *A* is the corresponding cross-sectional area perpendicular to the centerline.

### 2.5. Intraobserver Reproducibility Assessment

Twenty patients were randomly selected for intraobserver reproducibility analysis. All the measurements were repeated by the same observer after a 4-week interval while the observer was blinded to the initial results. Intraclass correlation coefficients (ICCs) and 95% confidence intervals were calculated using a two-way mixed-effects model with absolute agreement.

### 2.6. KD Size Groups

Because no universally accepted criteria distinguish aneurysmal from nonaneurysmal KD, patients were stratified by D_KDmax_ rather than by the presence or absence of an aneurysm. The 55 mm cutoff was selected from the threshold cited by the EACTS/ESVS consensus for considering repair [15], and D_KDmax_ was measured according to Idrees et al. [17]. Patients were classified as D_KDmax_ < 55 mm or D_KDmax_ ≥ 55 mm.

### 2.7. Statistical Analysis

Continuous variables are presented as mean ± standard deviation (*SD*), and categorical variables as counts and percentages. Normality was assessed using the Shapiro–Wilk test. Between-group comparisons used the independent-samples t test for normally distributed variables and the Mann–Whitney *U* test otherwise. Categorical variables were compared using the χ^2^ test or Fisher’s exact test, as appropriate.

Age associations for each morphological outcome were evaluated using sex-adjusted linear regression. Group differences in morphology were evaluated using analysis of covariance adjusted for age and sex. Pearson correlation coefficients were calculated for D_KDmax_ versus D_10_ and for D_10_ versus L_4_ and L_5_. Separate multivariable linear regression models examined associations between D_10_ and D_1_–D_8_, with each non-KD diameter entered as the dependent variable and age and sex as covariates. Two additional models used L_4_ and L_5_ as the respective dependent variables, with D_10_ as the principal explanatory variable and age and sex as covariates. Unstandardized coefficients (*B*), 95% confidence intervals, and standardized coefficients (*β*) are reported.

To evaluate the robustness of the principal geometric associations, the regression models for D_7_, L_4_, and L_5_ were repeated using D_KDmax_ in place of D_10_ as the measure of KD size. The primary D_10_ models were also repeated with additional adjustment for height. In a separate sensitivity analysis, patients were reclassified using an alternative D_KDmax_ cutoff of 50 mm, and differences in D_7_, L_4_, and L_5_ were evaluated using analysis of covariance adjusted for age and sex. Effect directions, magnitudes, and 95% CIs were compared with those from the primary analyses.

To address multiple testing, Benjamini–Hochberg false discovery rate (FDR) correction was applied separately within the prespecified families of non-KD-related aortic diameters, KD-related aortic diameters, and aortic lengths for the age and group analyses, and across the D_10_ models for D_1_–D_8_. An FDR-adjusted *q* value < 0.05 was considered significant. All the other tests were two-sided, with *p* < 0.05 considered significant. Missing data were not imputed; available-case analyses were performed. Analyses were conducted using IBM SPSS Statistics (version 22.0; IBM Corp., Armonk, NY, USA).

## 3. Results

### 3.1. Baseline Characteristics

A total of 65 adults with RAA-KD were included: 38 with D_KDmax_ < 55 mm and 27 with D_KDmax_ ≥ 55 mm (Table 1). The mean age was 55.2 ± 11.3 years. Pneumonia was recorded in 24 patients (36.9%), dysphagia in 10 (15.4%), and dyspnea in 9 (13.8%). All 65 patients were managed nonoperatively, and none underwent open surgical, hybrid, or endovascular repair during the study period. Hypertension, diabetes mellitus, ALSA stenosis, vertebral artery variants, thyroid hemiagenesis, and an aberrant left brachiocephalic vein were uncommon.

Patients with D_KDmax_ ≥ 55 mm were older than those with D_KDmax_ < 55 mm (59.0 ± 11.9 vs. 52.5 ± 10.1 years; *p* = 0.021), less frequently female (18.5% vs. 42.1%; *p* = 0.045) and more frequently had aortic arch calcification (70.4% vs. 42.1%; *p* = 0.024). No statistically significant between-group differences were observed in height, clinical presentation, comorbidities, or other associated vascular and anatomical findings (Table 1).

### 3.2. Intraobserver Reproducibility Results

Intraobserver reproducibility was good to excellent, with ICCs ranging from 0.779 to 0.978 (Table S1).

### 3.3. Age-Associated Thoracic Aortic Morphology

**Figure 2:**
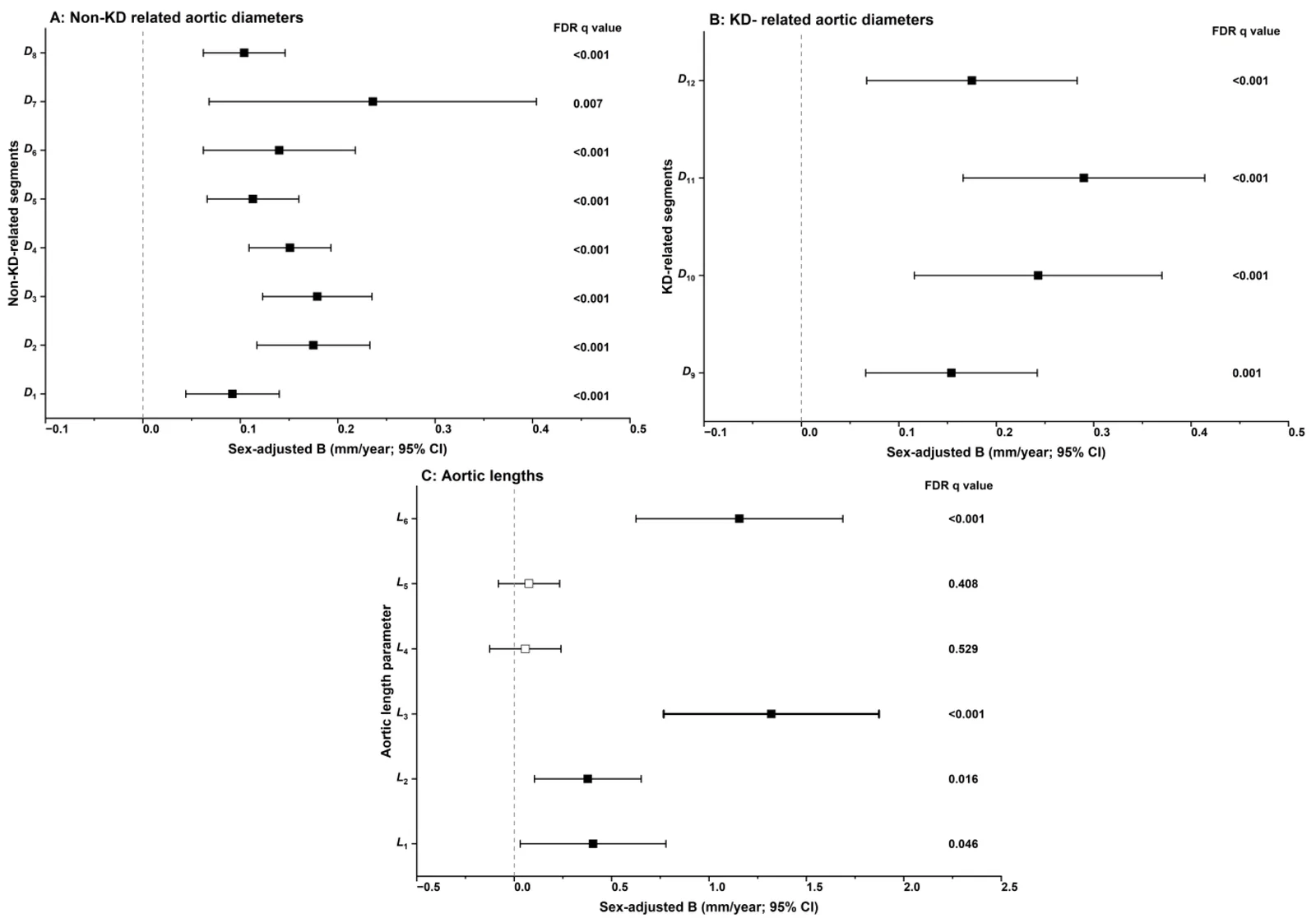
Associations between age and thoracic aortic morphology after adjustment for sex: (**A**) Associations between age and non-KD-related aortic diameters. (**B**) Associations between age and KD-related aortic diameters. (**C**) Associations between age and aortic lengths. Squares represent regression coefficients for age; filled squares indicate associations that remained statistically significant after FDR correction (*q* < 0.05), whereas open squares indicate nonsignificant associations (*q* ≥ 0.05). Horizontal bars indicate 95% confidence intervals, and the dashed vertical line represents the null value (B = 0). Regression models were adjusted for sex. False discovery rate (FDR) *q* values were calculated using the Benjamini–Hochberg procedure.

In sex-adjusted linear regression, all non-KD-related and KD-related aortic diameters were positively associated with age and remained significant after FDR correction (all *q* < 0.05; Figure 2). L_1_, L_2_, L_3_, and L_6_ also increased with age (*q* = 0.046, *q* = 0.016, *q* < 0.001, and *q* < 0.001, respectively). In contrast, neither L_4_ nor L_5_ was associated with age (*q* = 0.529 and *q* = 0.408, respectively). Thus, age was associated with generalized radial enlargement and elongation of the major thoracic aortic segments, without detectable differences in aortic isthmus or KD length.

### 3.4. Morphological Differences by D_KDmax_ Group

**Figure 3:**
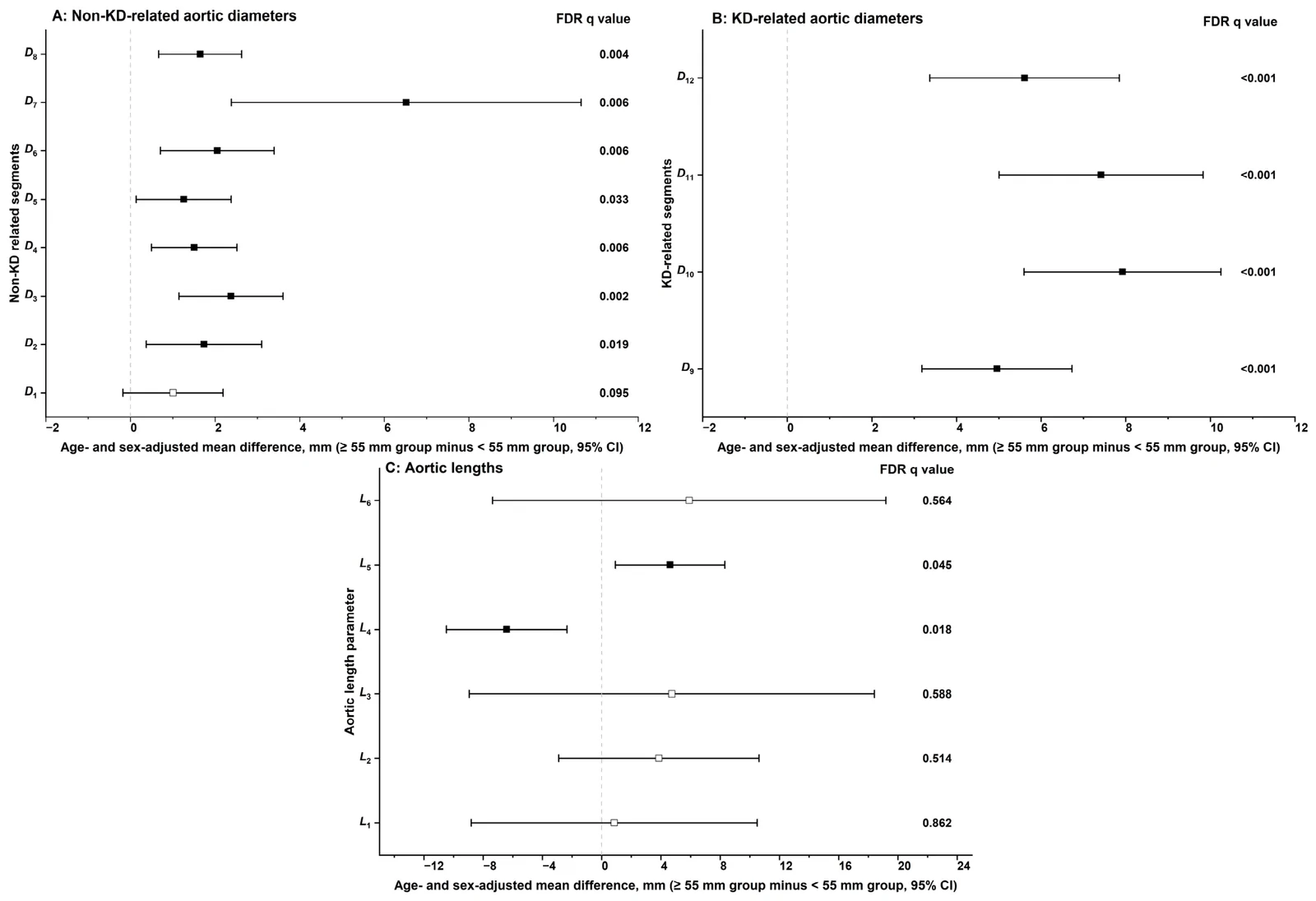
Age- and sex-adjusted differences in thoracic aortic morphology between patients with D_KDmax_ ≥ 55 mm and D_KDmax_ < 55 mm: (**A**) Non-KD-related aortic diameters. (**B**) KD-related diameters. (**C**) Aortic lengths. Squares indicate adjusted mean differences estimated using ANCOVA after adjustment for age and sex. Horizontal lines represent 95% confidence intervals and the dashed vertical line represents the null value (adjusted mean difference = 0). Open squares indicate nonsignificant comparisons after FDR correction (*q* ≥ 0.05), whereas filled squares indicate significant differences (*q* < 0.05). Positive values indicate larger measurements in the D_KDmax_ ≥ 55 mm group. False discovery rate (FDR) was controlled using the Benjamini–Hochberg procedure.

All non-KD and KD-related aortic diameters were larger in the D_KDmax_ ≥ 55 mm group in unadjusted analyses (Table 2 and Figure 3). After adjustment for age and sex and FDR correction, D_2_–D_8_ remained significantly larger (all *q* ≤ 0.033), whereas D_1_ did not (*q* = 0.095). All KD-related diameters (D_9_–D_12_) remained larger in the ≥55 mm group (all *p* < 0.001).

For longitudinal morphology, the ≥55 mm group had longer L_2_, L_3_, and L_6_, a shorter L_4_, and a longer L_5_ in unadjusted analyses; L_1_ did not differ. After adjustment for age and sex and FDR correction, differences in L_1_–L_3_ and L_6_ were not significant. L_4_ remained shorter (*q* = 0.018), whereas L_5_ remained longer (*q* = 0.045) in the ≥ 55 mm group.

### 3.5. Geometric Associations with KD Enlargement

**Figure 4:**
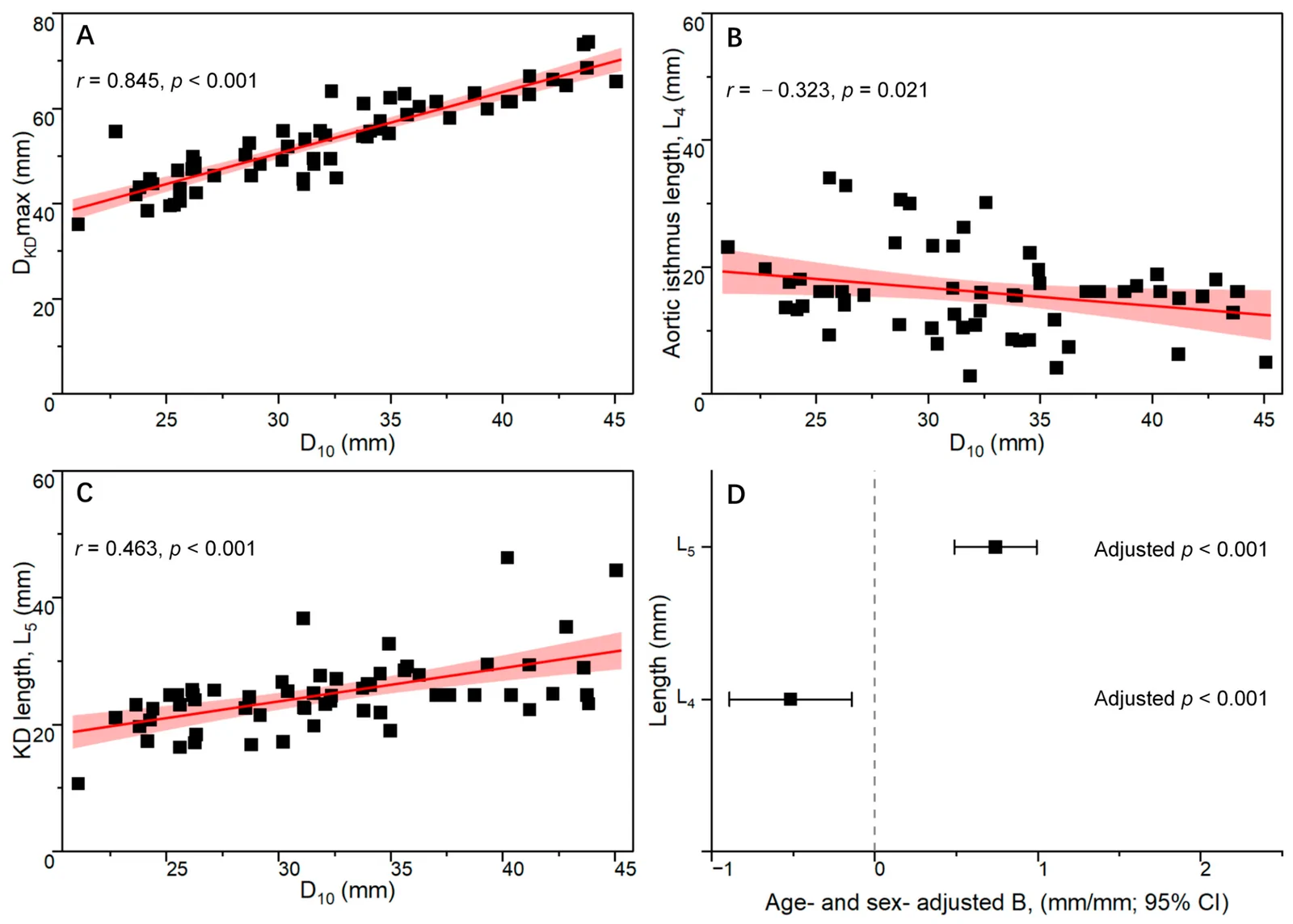
Relationship between D_10_ and localized geometric remodeling in patients with right-sided aortic arch and Kommerell diverticulum: (**A**) Correlation between D_10_ and the conventional maximal Kommerell diverticulum diameter (D_KDmax_). (**B**) Correlation between D_10_ and aortic isthmus length (L_4_). (**C**) Correlation between D_10_ and Kommerell diverticulum length (L_5_). (**D**) Multivariable linear regression analyses of L_4_ and L_5_ with D_10_ after adjustment for age and sex. Squares indicate regression coefficients, horizontal bars represent 95% confidence intervals, and the dashed vertical line indicates the null value (B = 0).

D_KDmax_ correlated strongly with D_10_ (r = 0.845; *p* < 0.001), demonstrating close correspondence between the conventional maximal KD diameter and the centerline-derived equivalent diameter at maximal cross-sectional area (Figure 4A). This association does not establish interchangeability because the two measurements represent different geometric constructs.

When KD size was analyzed continuously, D_10_ was positively associated with every non-KD aortic diameter (D_1_–D_8_) after adjustment for age and sex, and all associations remained significant after FDR correction (all *q* ≤ 0.020; Figure S1). The largest coefficient was observed for D_7_ (B = 0.803 mm/mm; *q* < 0.001), indicating that the association with KD size was most pronounced in the adjacent proximal descending aorta.

D_10_ was inversely correlated with aortic isthmus length (L_4_; r = −0.323; *p* = 0.021) and positively correlated with KD length (L_5_; r = 0.463; *p* < 0.001) (Figure 4B,C). In separate multivariable models adjusted for age and sex, D_10_ remained inversely associated with L_4_ (B = −0.548 mm/mm; 95% CI, −0.828 to −0.268; standardized β = −0.505; *p* < 0.001) and positively associated with L_5_ (B = 0.639 mm/mm; 95% CI, 0.412–0.866; standardized β = 0.679; *p* <0.001) (Figure 4D and Table 3).

### 3.6. Sensitivity Analysis

The principal geometric associations remained consistent across sensitivity analyses. When D_KDmax_ was substituted for D_10_ as the measure of KD size, larger D_KDmax_ remained positively associated with D_7_ (B = 0.412; 95% CI, 0.221–0.603; standardized β = 0.536; *p* < 0.001), inversely associated with L_4_ (B = −0.370; 95% CI, −0.553 to −0.187; standardized β = −0.516; *p* < 0.001), and positively associated with L_5_ (B = 0.301; 95% CI, 0.134–0.468; standardized β = 0.484; *p* < 0.001). Additional adjustment for height did not materially alter the associations of D_10_ with D_7_ (B = 0.854; 95% CI, 0.446–1.262; standardized β = 0.709; *p* < 0.001), L_4_ (B = −0.589; 95% CI, −0.982 to −0.195; standardized β = −0.536; *p* = 0.005), or L_5_ (B = 0.817; 95% CI, 0.460–1.174; standardized β = 0.736; *p* < 0.001) (Table S2).

Reclassification using an alternative D_KDmax_ cutoff of 50 mm produced a pattern consistent with the primary 55 mm analysis (Table S3). After adjustment for age and sex, the ≥50 mm group had a larger D_7_ (adjusted mean difference, 4.7 mm; 95% CI, 0.78–8.6; *p* = 0.020), a shorter L_4_ (adjusted mean difference, −6.9 mm; 95% CI, −10.7 to −3.1; *p* < 0.001), and a longer L_5_ (adjusted mean difference, 4.8 mm; 95% CI, 1.3–8.3; *p* = 0.008) than the <50 mm group. These findings support the robustness of the principal regional morphological associations to the selected KD-size measure, additional adjustment for height, and the choice of D_KDmax_ cutoff.

## 4. Discussion

### 4.1. Principal Findings

This study provides a comprehensive three-dimensional, centerline-based assessment of radial and longitudinal thoracic aortic morphology in adults with RAA-KD. Four principal findings emerged. First, increasing age was associated with generalized enlargement of KD-related and non-KD aortic segments and elongation of the ascending aorta, arch, and descending thoracic aorta. Second, after age and sex adjustment and FDR correction, patients with D_KDmax_ ≥ 55 mm had larger D_2_–D_8_ and D_9_–D_12_; continuous analyses confirmed positive associations between D_10_ and all non-KD diameters, strongest at D_7_. Third, larger KD size was associated with a shorter aortic isthmus and a longer diverticulum, independent of age and sex. Finally, this geometry may constrain the proximal sealing zone for TEVAR, supporting comprehensive assessment of the adjacent thoracic aorta rather than reliance on maximal KD diameter alone.

### 4.2. Value of Three-Dimensional Measurements in RAA-KD

Morphological assessment of RAA-KD is challenging because of its complex three-dimensional configuration. The approaches described by Ota and Idrees remain clinically important, but they focus mainly on diverticular size and provide limited information about the spatial relationship between the KD and adjacent thoracic aorta [17,18]. Centerline-based analysis permits cross-sectional measurements perpendicular to the vessel trajectory and longitudinal measurements along the aorta, reducing errors related to tortuosity and oblique imaging planes [24]. This unified framework allowed focal KD enlargement and changes in adjoining and more remote thoracic aortic segments to be assessed together.

The strong correlation between D_KDmax_ and D_10_ supports the internal consistency of the measurements while underscoring their distinct roles. D_KDmax_ is a clinically familiar distance from the contralateral aortic wall to the diverticular apex and was used for patient stratification. D_10_ is an equivalent diameter derived from maximal cross-sectional area perpendicular to the centerline. They should therefore be considered complementary, not interchangeable.

### 4.3. Age-Associated Thoracic Aortic Remodeling

Age-associated morphological differences extended beyond the diverticulum. All non-KD and KD-related diameters increased with age after sex adjustment and FDR correction, as did L_1_, L_2_, L_3_, and L_6_. This pattern resembles the radial enlargement and longitudinal elongation described in the general thoracic aorta [26,27] and suggests that similar age-associated processes may occur in the congenitally variant thoracic aorta of adults with RAA-KD.

Longitudinal evidence in patients with RAA-KD remains limited and has largely focused on changes in diverticular size. Erben et al. reported mean growth rates of 1.45 ± 0.39 mm/year for KD diameter and 2.29 ± 0.47 mm/year for the distance from the diverticular apex to the opposite aortic wall among patients followed for a mean of 31.7 months [12]. Hale et al. found that absolute KD diameter was positively associated with age, whereas KD diameter indexed to the descending aortic diameter was not, suggesting that age-associated KD enlargement may occur proportionally with enlargement of the surrounding aorta [13]. A recent observational cohort similarly evaluated KD dimensions and growth in patients with RAA and ALSA [8]. Our findings extend this literature by demonstrating age associations across multiple radial and longitudinal thoracic aortic measurements. Nevertheless, because our analysis was cross-sectional, it cannot establish individual growth rates.

Neither L_4_ nor L_5_ was associated with age, yet both were associated with D_10_ after multivariable adjustment. This contrast suggests that the longitudinal geometry of the KD complex may reflect regional KD-associated remodeling rather than generalized age-associated elongation. Because the study was cross-sectional, however, the direction and temporal sequence of these associations cannot be determined.

### 4.4. Localized Geometric Remodeling Associated with KD Enlargement

Morphological differences associated with larger KD size extended beyond the diverticulum itself. D_2_–D_8_ remained larger in the D_KDmax_ ≥ 55 mm group after age and sex adjustment and FDR correction, whereas D_1_ did not. Although D_1_ was not significantly different in the dichotomous comparison, continuous regression analysis demonstrated a modest positive association between D_10_ and D_1_. This difference may reflect the greater statistical efficiency of retaining KD size as a continuous variable, whereas dichotomization at the 55 mm threshold results in information loss and reduced statistical power.

Continuous models showed that D_10_ was positively associated with all non-KD diameters, with the largest coefficient at D_7_. These complementary analyses suggest broader differences in thoracic aortic caliber, particularly in the proximal descending aorta adjacent to KD. These findings were further supported by sensitivity analyses using D_KDmax_ in place of D_10_, additional adjustment for height, and an alternative D_KDmax_ cutoff of 50 mm. The consistency of the results across these analyses indicates that the principal regional morphological pattern was robust to the selected KD-size measure, additional height adjustment, and the choice of dichotomization threshold.

Longitudinal geometry showed a more regional pattern. The ≥55 mm group had a shorter L_4_ and a longer L_5_ after adjustment and FDR correction; in continuous models, each 1 mm increase in D_10_ was associated with a 0.548 mm decrease in L_4_ and a 0.639 mm increase in L_5_. These reciprocal associations support coordinated local remodeling in which larger KD size accompanies longitudinal extension of the diverticulum and shortening of the intervening aortic isthmus.

The mechanisms underlying these associations remain uncertain. Ultrastructural observations in KD specimens have described medial degeneration and disruption of elastic tissue, suggesting regional structural vulnerability of the aortic wall [28]. Altered curvature, flow, and wall stress at the distal arch–isthmus–KD junction may also contribute, but this explanation remains hypothetical because histologic, biomechanical, and hemodynamic assessments were not performed. Longitudinal imaging with patient-specific flow and wall-stress analyses will be needed to clarify the sequence and mechanism of these changes.

### 4.5. Clinical Implications for Treatment Planning

Although many asymptomatic KDs follow an indolent course, aortic dissection and rupture have been reported. Austin and Wolfe identified rupture in 6 of 32 cases [11], whereas Cinà et al. reported 12 dissections and two ruptures among 32 published cases [1]. These historical estimates were derived from selected case reports and surgical populations and should not be interpreted as population-level incidence rates. In a contemporary cohort, Erben et al. observed one untreated rupture among 66 patients during a mean follow-up of 31.7 months [12], suggesting that many incidentally detected KDs remain stable or enlarge slowly during intermediate-term follow-up. Nevertheless, surveillance and treatment decisions should consider symptoms, associated aortic pathology, interval growth, and anatomical characteristics in addition to absolute KD size.

Management recommendations rely on symptoms and KD size, but thresholds and measurement methods remain inconsistent. The 2019 EACTS/ESVS consensus states that repair should be considered for KD measuring ≥5.5 cm, whereas the 2022 ACC/AHA guideline considers repair reasonable when the KD orifice exceeds 3.0 cm or the combined diameter from the diverticular apex to the opposite aortic wall exceeds 5.0 cm [15,29]. These differences reinforce the need to report the measurement method explicitly. Observational data also suggest that smaller asymptomatic KDs may follow an indolent short-term course, supporting individualized surveillance and multidisciplinary decision-making [7,8,12].

Preoperative CTA assessment should not be limited to D_KDmax_. Proximal descending aortic caliber, aortic isthmus length, and diverticular longitudinal extent may affect device selection and proximal sealing-zone planning [30]. A healthy proximal sealing zone of approximately 20 mm is commonly used as a planning benchmark for conventional TEVAR, although the required length varies among devices and may be greater in the aortic arch [16]. Device-specific instructions for use, arch angulation, aortic diameter, and landing-zone wall quality remain decisive. When an adequate native sealing zone is unavailable, supra-aortic debranching, hybrid repair, or other techniques that extend the proximal landing zone may be considered [17,31,32].

In this cohort, mean L_4_ was ≤20 mm in patients with D_KDmax_ ≥ 55 mm. Because L_4_ represents the native aortic segment between the distal edge of the RSA and the proximal edge of the KD, conventional TEVAR using this segment as the proximal sealing zone may be anatomically unsuitable in some patients. Supra-aortic branch reconstruction, hybrid repair, fenestrated or branched devices, or alternative proximal landing strategies may be required. However, L4 alone does not determine procedural suitability; curvature, calcification, thrombus, wall quality, access anatomy, and device instructions for use must also be considered. Because procedural outcomes were unavailable, these findings should be interpreted as anatomical observations relevant to treatment planning rather than evidence favoring any specific repair strategy.

### 4.6. Limitations

This study has several limitations. First, its retrospective, single-center design introduced potential referral and selection bias. Second, the sample was modest, although RAA-KD is rare. Third, the cross-sectional design precluded assessment of individual remodeling trajectories and could not determine whether KD enlargement precedes, accompanies, or follows changes in adjacent aortic geometry. Fourth, the absence of a normal left-arch control group or an RAA group without KD prevents separation of KD-specific features from those attributable to the right-sided arch configuration. Fifth, body-size data were incomplete, precluding consistent indexing of aortic measurements to BSA; residual confounding by body size may remain despite adjustment for sex. Sixth, equivalent diameters were derived from cross-sectional areas and may not fully describe irregular geometry. Seventh, histopathologic, biomechanical, and hemodynamic data were unavailable, and quantitative arch curvature was not assessed. Its potential association with KD progression or adverse aortic events requires evaluation in longitudinal studies using standardized three-dimensional methods. Finally, because all included patients were managed nonoperatively and standardized longitudinal CTA examinations were unavailable, treatment-related aortic remodeling and post-intervention outcomes could not be assessed.

## 5. Conclusions

Adults with RAA-KD demonstrated two overlapping morphological patterns: generalized age-associated enlargement and elongation of the major thoracic aortic segments, and localized KD-associated remodeling characterized by aortic enlargement extending beyond the diverticulum, a shorter aortic isthmus, and a longer diverticulum. These cross-sectional findings support systematic centerline-based evaluation of the adjacent thoracic aorta rather than reliance on maximal KD diameter alone. The potential implications of these anatomical features for TEVAR planning require validation in longitudinal cohorts incorporating procedural and clinical outcomes.

## Figures and Tables

**Table 1:** Baseline characteristics of patients with right-sided aortic arch and Kommerell diverticulum according to maximal Kommerell diverticulum diameter (D_KDmax_).

		Overall (*n* = 65)	D_KDmax_ < 55 mm (*n* = 38)	D_KDmax_ ≥ 55 mm (*n* = 27)	*p* Value
Age (years)		55.2 ± 11.3	52.5 ± 10.1	59.0 ± 11.9	0.021 *
Female sex		21 (32.3%)	16 (42.1%)	5 (18.5%)	0.045 *
Height (cm) ^a^		164.5 ± 7.0	164.8 ± 6.7	164.2 ± 7.4	0.804
Clinic presentation	dysphagia	10 (15.4%)	4 (10.5%)	6 (22.2%)	0.297
	dyspnea	9 (13.8%)	4 (10.5%)	5 (18.5%)	0.472
	pneumonia	24 (36.9%)	12 (31.6%)	12 (44.4%)	0.310
Comorbidities	Hypertension	6 (9.2%)	2 (5.3%)	4 (14.8%)	0.224
	Diabetes mellitus	1 (1.5%)	0	1 (3.7%)	0.415
Associated anomalies	Stenosis of ALSA	3 (4.6%)	2 (5.3%)	1 (3.7%)	1.0
	Aortic arch calcification	35 (53.8%)	16 (42.1%)	19 (70.4%)	0.024 *
	Vertebral artery variations	3 (4.6%)	2 (5.3%)	1 (3.7%)	1.0
	thyroid hemiagenesis	2 (3.1%)	2 (5.3%)	0	0.507
	aberrant left brachiocephalic vein	3 (4.6%)	2 (5.3%)	1 (3.7%)	1.0

Data are presented as mean ± SD or n (%). Comparisons were performed using the independent-samples t test or Mann–Whitney U test for continuous variables and χ^2^ test or Fisher’s exact test for categorical variables. * *p* < 0.05. ^a^ Height data were available for 35 patients. KD: Kommerell diverticulum; ALSA: aberrant left subclavian artery; D_KDmax_: conventional maximal Kommerell diverticulum diameter; SD: standard deviation.

**Table 2:** Comparison of thoracic aortic morphology between patients with D_KDmax_ < 55 mm and D_KDmax_ ≥ 55 mm.

		D_KDmax_ < 55 mm (*n* = 38)	D_KDmax_ ≥ 55 mm (*n* = 27)	Unadjusted *p* Value	Age- and Sex-Adjusted *p* Value	FDR *q* Value
Non-KD-related aortic diameters (mm)	D_1_	22.3 ± 2.3	24.6 ± 2.0	**0.001**	0.095	0.095
	D_2_	23.7 ± 3.0	26.9 ± 2.6	**<0.001**	**0.014**	**0.019**
	D_3_	22.4 ± 2.8	26.0 ± 2.7	**<0.001**	**<0.001**	**0.002**
	D_4_	21.0 ± 2.3	23.7 ± 2.3	**<0.001**	**0.004**	**0.006**
	D_5_	19.9 ± 2.4	22.0 ± 2.1	**<0.001**	**0.029**	**0.033**
	D_6_	20.1 ± 2.7	23.3 ± 2.5	**<0.001**	**0.003**	**0.006**
	D_7_	27.9 ± 6.5	35.7 ± 6.2	**<0.001**	**0.003**	**0.006**
	D_8_	16.8 ± 2.1	19.7 ± 2.0	**<0.001**	**0.001**	**0.004**
KD-related aortic diameters (mm)	D_9_	21.2 ± 3.6	27.4 ± 3.2	**<0.001**	**<0.001**	**<0.001**
	D_10_	28.2 ± 3.5	37.5 ± 5.1	**<0.001**	**<0.001**	**<0.001**
	D_11_	23.1 ± 4.6	32.1 ± 5.2	**<0.001**	**<0.001**	**<0.001**
	D_12_	19.7 ± 4.0	26.4 ± 4.4	**<0.001**	**<0.001**	**<0.001**
Aortic lengths (mm)	L_1_	56.3 ± 15.1	59.6 ± 14.7	0.459	0.862	0.862
	L_2_	44.5 ± 10.9	51.6 ± 12.4	**0.034**	0.257	0.514
	L_3_	213.7 ± 25.5	230.2 ± 28.9	**0.036**	0.49	0.588
	L_4_	17.8 ± 7.7	13.7 ± 6.3	**0.048**	**0.003**	**0.018**
	L_5_	22.8 ± 4.9	27.2 ± 7.2	**0.010**	**0.015**	**0.045**
	L_6_	172.5 ± 23.2	188.8 ± 27.3	**0.023**	0.376	0.564

D_1_–D_12_ denote diameters measured at predefined thoracic aortic landmarks: sino-tubular junction (D_1_), mid ascending aorta (D_2_), distal ascending aorta (D_3_), proximal aortic arch (D_4_), distal aortic arch (D_5_), aortic isthmus (D_6_), proximal descending thoracic aorta (2 cm distal to the distal margin of the Kommerell diverticulum; D_7_), diaphragmatic hiatus (D_8_), proximal KD (D_9_), maximal KD cross-sectional level (D_10_), distal KD (D_11_), and the orifice of the KD (D_12_). L_1_–L_6_ denote the lengths of the ascending aorta, aortic arch, descending thoracic aorta, aortic isthmus, Kommerell diverticulum, and descending thoracic aorta distal to the KD, respectively. Data are presented as mean ± SD. Adjusted *p* values were obtained using analysis of covariance (ANCOVA) with age and sex as covariates. Multiple comparisons were corrected using the Benjamini–Hochberg false discovery rate (FDR) procedure. Bold values indicate statistical significance (*p* < 0.05 or FDR-adjusted *q* < 0.05).

**Table 3:** Multivariable linear regression analyses of aortic isthmus length and KD length.

Outcome	Predictor	B	95% CI	Standardized β	*p* Value
Aortic isthmus length, L_4_	D_10_	−0.548	−0.828 to −0.268	−0.505	<0.001
	Age	0.169	0.026 to 0.313	0.291	0.021
	Sex	−5.562	−8.850 to −2.273	−0.395	0.001
KD length, L_5_	D_10_	0.639	0.412 to 0.866	0.679	<0.001
	Age	−0.083	−0.199 to 0.033	−0.164	0.159
	Sex	2.075	−0.583 to 4.734	0.170	0.124

Separate multivariable linear regression models were fitted for aortic isthmus length (L_4_) and KD length (L_5_). Each model included D_10_, age, and sex. B represents the unstandardized regression coefficient. For D_10_, B is expressed in mm/mm; for age, in mm/year; and for sex, as the adjusted mean difference between the coded sex categories. CI, confidence interval; KD, Kommerell diverticulum. Sex was coded as 0 = female and 1 = male.

## Data Availability

The data supporting the findings of this study are available from the corresponding author upon reasonable request, subject to institutional and privacy restrictions.

## Supplementary Materials

The following supporting information can be downloaded at https://www.mdpi.com/article/10.3390/jcdd13090427/s1. Figure S1: Associations between D_10_ and non-KD-related aortic diameters after adjustment for age and sex; Table S1: Intraobserver reproducibility of morphologic measurements; Table S2: Sensitivity Analyses of Associations between KD Size and Key Thoracic Aortic Measurements; Table S3: Sensitivity analysis using an alternative D_KDmax_ cutoff of 50 mm.
