# Supplementary material for: Three-Dimensional Thoracic Aortic Remodeling Associated with Kommerell Diverticulum Enlargement in Adults with a Right-Sided Aortic Arch: A Quantitative CTA Study
Source: J Cardiovasc Dev Dis. 2026 Sep 1;13(9):427. doi: 10.3390/jcdd13090427 (PMC13607531; doi:10.3390/jcdd13090427)
Supplement: Supplementary file 1 [file jcdd-13-00427-s001.zip › Supplementary Figure S1.pdf]

Supplementary Figure S1. Associations between D_{10} and non-KD-related thoracic aortic diameters after adjustment for age and sex.

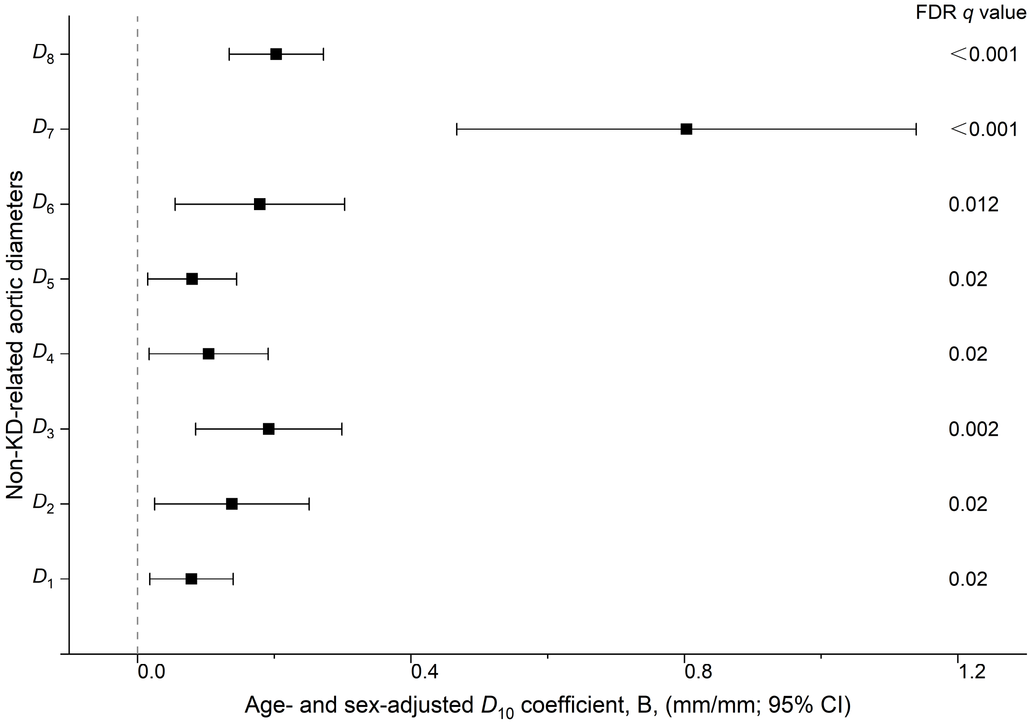

Squares represent regression coefficients for D_{10} obtained from multivariable linear regression models adjusted for age and sex. Horizontal bars indicate 95% confidence intervals. The dashed vertical line represents the null value (B = 0). False discovery rate (FDR) was controlled using the Benjamini–Hochberg procedure.
