# Supplementary material for: Three-Dimensional Thoracic Aortic Remodeling Associated with Kommerell Diverticulum Enlargement in Adults with a Right-Sided Aortic Arch: A Quantitative CTA Study
Source: J Cardiovasc Dev Dis. 2026 Sep 1;13(9):427. doi: 10.3390/jcdd13090427 (PMC13607531; doi:10.3390/jcdd13090427)
Supplement: Supplementary file 1 [file jcdd-13-00427-s001.zip › Supplementary Table S1.pdf]

Table S1. Inraobserver reproducibility of morphologic measurements

Measurement category	Parameter	ICCs (95% CI)
Non-KD-related aortic diameters	D_1	0.788 (0.519-0.915)
	D_2	0.958 (0.898-0.983)
	D_3	0.779 (0.502-0.911)
	D_4	0.962 (0.904-0.985)
	D_5	0.948 (0.874-0.979)
	D_6	0.963 (0.908-0.985)
	D_7	0.967 (0.924-0.986)
	D_8	0.970 (0.927-0.988)
KD-related aortic diameters	D_9	0.961 (0.908-0.984)
	D_{10}	0.950 (0.877-0.980)
	D_{11}	0.963 (0.908-0.985)
	D_{12}	0.878 (0.724-0.949)
	D_{KDmax}	0.951 (0.881-0.980)
Aortic lengths	L_1	0.782 (0.529-0.908)
	L_2	0.970 (0.926-0.988)
	L_3	0.955 (0.890-0.980)
	L_4	0.978 (0.946-0.991)
	L_5	0.949(0.879-0.979)
	L_6	0.957 (0.899-0.982)

ICCs were calculated using a two-way mixed-effects model with absolute agreement for single measurements. Twenty randomly selected patients were measured twice by the same observer at a 4-week interval. ICC, intraclass correlation coefficient; CI, confidence interval; KD, Kommerell diverticulum.
