# Supplementary material for: Three-Dimensional Thoracic Aortic Remodeling Associated with Kommerell Diverticulum Enlargement in Adults with a Right-Sided Aortic Arch: A Quantitative CTA Study
Source: J Cardiovasc Dev Dis. 2026 Sep 1;13(9):427. doi: 10.3390/jcdd13090427 (PMC13607531; doi:10.3390/jcdd13090427)
Supplement: Supplementary file 1 [file jcdd-13-00427-s001.zip › Supplementary Table S2.pdf]

Table S2. Sensitivity Analyses of Associations between KD Size and Key Thoracic Aortic Measurements

Outcome	Analysis	KDsizemeasure	Covariates	*B*	95%CI	Standardized*β*	*p*
Proximal descending aorta (D_7)	main	D_{10}	Age + sex	0.746	0.475 to 1.017	0.640	＜0.001
	Alternative measurement	D_{KDmax}	Age + sex	0.412	0.221 to 0.603	0.536	＜0.001
	Height-asjusted	D_{10}	Age + sex + height	0.854	0.446 to 1.262	0.709	＜0.001
Aortic isthmus (L_4)	main	D_{10}	Age + sex	-0.548	-0.828 to -0.248	-0.505	＜0.001
	Alternative measurement	D_{KDmax}	Age + sex	-0.370	-0.553 to -0.187	-0.516	＜0.001
	Height-asjusted	D_{10}	Age + sex + height	-0.589	-0.982 to -0.195	-0.536	0.005
KD length (L_5)	main	D_{10}	Age + sex	0.639	0.412 to 0.866	0.679	＜0.001
	Alternative measurement	D_{KDmax}	Age + sex	0.301	0.134 to 0.468	0.484	＜0.001
	Height-asjusted	D_{10}	Age + sex + height	0.817	0.460 to 1.174	0.736	＜0.001

B represents the unstandardized regression coefficient. All outcomes and KD-size measures were expressed in millimeters; therefore, B is expressed as mm/mm. Standardized β is dimensionless. Each outcome was analyzed in a separate linear regression model. The main and alternative-measurement models were adjusted for age and sex, whereas the height-adjusted models were additionally adjusted for height.
