# Supplementary material for: Three-Dimensional Thoracic Aortic Remodeling Associated with Kommerell Diverticulum Enlargement in Adults with a Right-Sided Aortic Arch: A Quantitative CTA Study
Source: J Cardiovasc Dev Dis. 2026 Sep 1;13(9):427. doi: 10.3390/jcdd13090427 (PMC13607531; doi:10.3390/jcdd13090427)
Supplement: Supplementary file 1 [file jcdd-13-00427-s001.zip › Supplementary Table S3.pdf]

Table S3. Sensitivity analysis using an alternative D_{KDmax} cutoff of 50 mm

Outcome	<50mm,mean±SD	≥50mm,mean±SD	Adjusted mean difference	95%CI	*p*
D_7, (mm)	27.5 ±7.3	33.7 ± 6.3	4.7	0.78 to 8.6	0.020
L_4, (mm)	19.4 ± 8.0	13.7 ± 6.0	-6.9	-10.7 to -3.1	<0.001
L_5, (mm)	21.8 ± 5.1	26.7 ± 6.4	4.8	1.3 to 8.3	0.008

Values are presented as mean ± standard deviation. Adjusted mean differences were stimated using analysis of covariance with age and sex as covariates and are expressed as the D_{KDmax} ≥50-mm group minus the D_{KDmax} <50-mm group.
